# Association of 25-hydroxyvitamin D with cardiometabolic risk factors and metabolic syndrome: a mendelian randomization study

**DOI:** 10.1186/s12937-019-0494-7

**Published:** 2019-10-28

**Authors:** Chi Chen, Yi Chen, Pan Weng, Fangzhen Xia, Qin Li, Hualing Zhai, Ningjian Wang, Yingli Lu

**Affiliations:** Institute and Department of Endocrinology and Metabolism, Shanghai Ninth People’s Hospital, Shanghai JiaoTong University School of Medicine, Shanghai, 200011 China

**Keywords:** Vitamin D, Metabolic syndrome, Cardiometabolic risk factors, Mendelian randomization analysis

## Abstract

**Background:**

Low circulating vitamin D levels have been associated with increased risk of metabolic syndrome (MS) and cardiometabolic risk factors in multiple epidemiology studies. However, whether this association is causal is still unclear. We aimed to test whether genetically lowered vitamin D levels were associated with MS and its metabolic traits, using mendelian randomization (MR) methodology.

**Methods:**

Ten thousand six hundred fifty-five participants were enrolled from the SPECT-China study, which was performed in 23 sites in East China during 2014 to 2016. Using four single-nucleotide polymorphisms (SNPs) in the *DHCR7*, *CYP2R1*, *GC* and *CYP24A1* genes with known effects on 25(OH) D concentrations, we created a genetic risk score (GRS) as instrumental variable (IV) to estimate the effect of genetically lowered 25(OH) D on MS and cardiometabolic risk factors. MS was defined according to the International Diabetes Federation criteria.

**Results:**

Lower measured 25(OH)D levels were associated with MS (OR 0.921, 95% CI 0.888, 0.954) after multivariable adjustment. However, the MR-derived odds ratio of genetically determined 25(OH) D for risk of MS was 0.977 (95% CI 0.966, 1.030). The MR-derived estimates for raised fasting plasma glucose was 0.578 (95% CI 0.321, 0.980) per 10 nmol/L GRS_synthesis_ determined increase of 25(OH) D levels.

**Conclusions:**

We found no evidence that genetically determined reduction in 25(OH)D conferred an increased risk of MS and its metabolic traits. However, we created our GRS only on the basis of common variants, which represent limited amount of variance in 25(OH)D. MR studies using rare variants, and large-scale well-designed RCTs about the effect of vitamin D supplementation on MS are warranted to further validate the findings.

## Introduction

Metabolic syndrome (MS) is defined by an aggregation of cardiometabolic risk factors, including central obesity, hypertension, impaired glucose regulation, elevated triglycerides, and decreased high-density lipoprotein cholesterol that predispose the subject to developing multiple chronic diseases [1]. The prevalence of MS is increasing rapidly worldwide and it is estimated by the International Diabetes Federation that one quarter of the world’s adult population has MS [2]. In China, the prevalence of the MS was 33.9% (31.0% in men and 36.8% in women), which indicates that about 454 million adults were affected [3].

Vitamin D deficiency is also a global pandemic health problem and has been associated with MS and its metabolic traits in numerous cross-sectional studies [4–6]. However, it is still uncertain whether the observed association is causal or owing to confounding or reverse causation. In a dose-response meta-analysis by Sang Yhun Ju et al. [7], the association between lower Vitamin D status and risk of MS was only existed in cross-sectional studies, not in longitudinal studies. A most recent cross-sectional study found that adjusted odds ratios (OR) of MS in the fourth compared with the lowest quartile for serum 25(OH)D levels was 0.48 (95% CI 0.28–0.84) [8]. Clinical trials exploring the effect of vitamin D supplementation on cardiometabolic disorders were limited and failed to demonstrate a simultaneous protective effect [9–11]. Thus, the causality between vitamin D and MS and its components has not been confirmed in human beings.

Mendelian randomization (MR) uses genetic variants associated with an intermediate phenotype [in the present study, 25-hydroxyvitamin D (25(OH)D)] as instrumental variables (IVs) in non-experimental data to derive causal inferences about the effect of an exposure on an outcome [12]. In this study, if low 25(OH)D causally leads to MS, genetic variants associated with lower 25(OH)D should be associated with higher risk of MS and its metabolic traits [13]. Because genetic variants are randomly allocated at the time of gamete formation and assigned prior to outcome and confounders, MR studies are conceptually similar to randomized controlled trials (RCTs) and can overcome limitations in observational studies such as residual confounding and reverse causation [14]. Further, recent progresses in genotyping enable the application of MR approach in sample sizes that are not realistic for RCTs of vitamin D supplementation.

Using data from a large community-based sample of participants from the SPECT-China study (survey on prevalence in East China for metabolic diseases and risk factors), we investigated the association of 25(OH)D with cardiometabolic risk factors and MS. We inferred causality by using vitamin D genetic risk scores (VD_GRS) as IVs by MR analyses.

## Methods

### Study population

**Fig. 1:**
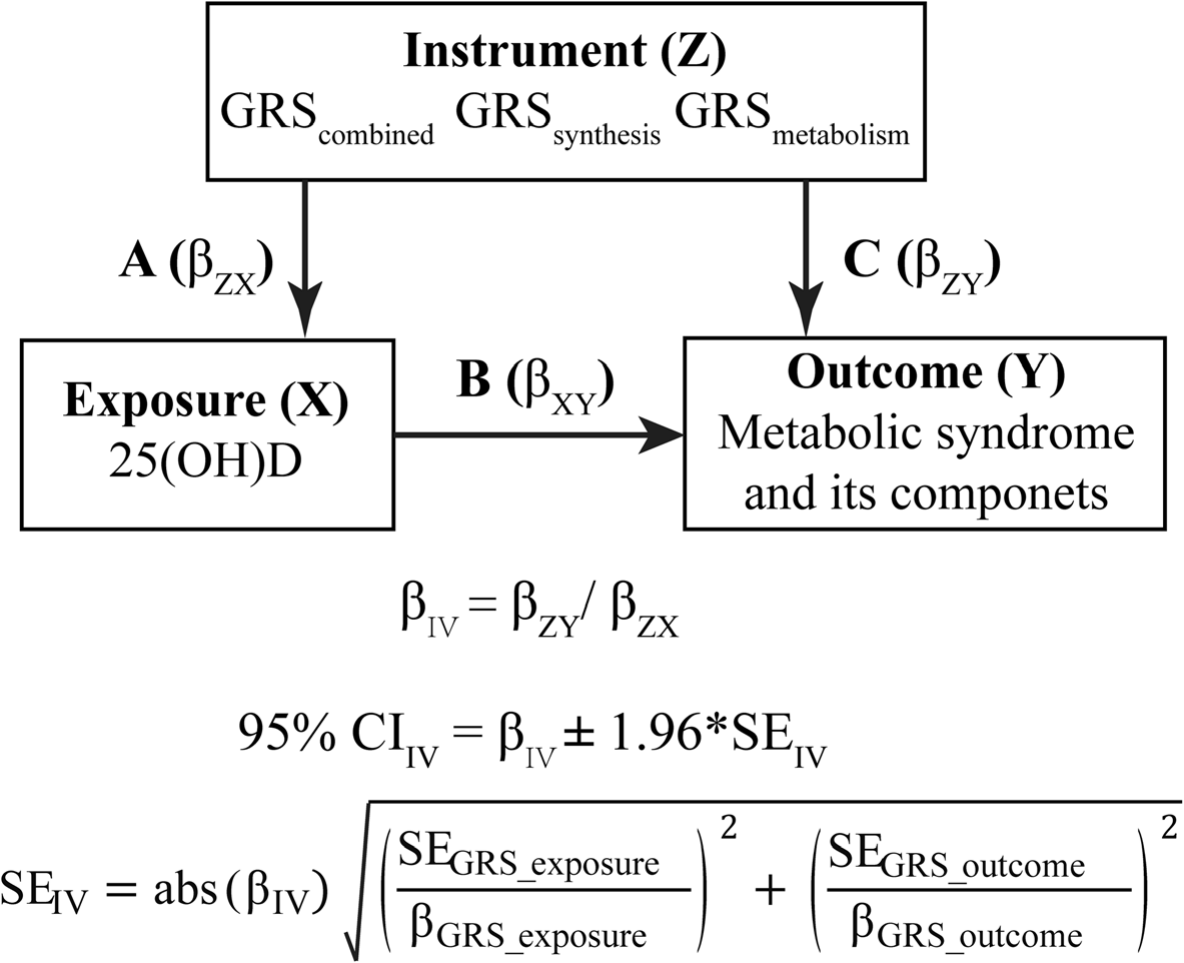
Study design and tested associations. GRS, genetic risk score; 25(OH)D, 25-hydroxyvitamin D; IV, instrumental variable. We assessed the association between vitamin D related genetic variants and 25(OH)D concentrations (**a**). Second, we measured the effects of genetic variants associated with 25(OH)D on the changes of metabolic components and MS risk (**c**). Third, we did observational assessments of the relation between 25(OH)D concentrations and present MS and its components (**b**)

Figure 1 shows the study design. We analyzed the observed associations between measured variables (residual confounding and reverse causation) and genetic associations between genotypes and measured variables (no residual confounding or reverse causation). First, we assessed the association between vitamin D related genetic variants and 25(OH)D concentrations (Fig. 1a). Second, we measured the effects of genetic variants associated with 25(OH)D on the changes of metabolic components and MS risk (Fig. 1c). Third, we did observational assessments of the relation between 25(OH)D concentrations and present MS and its components (Fig. 1b).

SPECT-China study population was used to describe the relationship between GRS and 25(OH)D levels, and to obtain IV results on the causal effect of 25(OH)D levels on MS and related traits. SPECT-China is an ongoing population-based cross-sectional study of 12,666 randomly selected community-dwelling individuals. Recruitment has been described in detail in previous papers [15–17]. Briefly, we used a stratified cluster sampling method to select a sample in the general population. The sampling process was stratified according to rural/urban area and economic development status. Random sampling was completed before data collection. From 2014 to 2016, the participants from 18 to 93 years in age were recruited from 23 sites in Shanghai, Zhejiang, Jiangsu, Anhui and Jiangxi provinces. Among them, 10,661 (84.2%) participants had genotype information of vitamin D single nucleotide polymorphisms (SNPs). Participants who had missing information on more than two SNP genotypes (*n* = 4) and 25(OH)D (*n* = 2) were excluded. The current study sample included 10,655 participants.

The Ethics Committee of Shanghai Ninth People’s Hospital, Shanghai JiaoTong University School of Medicine approved the study. The study was conducted in compliance with the principles of the 1975 Declaration of Helsinki as reflected in a priori approval by the appropriate institutional review committee. Informed written consent was obtained from all participants recruited in the study.

### Measurements

In each study site, the same staff performed all data collection. They were trained according to a standard protocol that made them familiar with the specific tools and methods used. The same trained staff used the standardized and validated questionnaire to collect information on socio-demographic information, medical and family history, general health, medication, etc.

Weight, height, waist circumference and BP were assessed by standard methods [18]. Body mass index (BMI) was calculated as weight in kilograms divided by height in meters squared. Current smoking was defined as having smoked at least 100 cigarettes in one’s lifetime and currently smoking cigarettes [18]. Each site was categorized into high and low economic status, comparing local gross domestic product per capita with the national one in 2013 [15].

Fasting plasma glucose (FPG), triglycerides (TG) and high density lipoprotein (HDL) were measured using the AU 680 (Beckman Coulter, Brea, USA). The 25(OH)D was detected using a chemiluminescence assay (ADVIA Centaur XP, Siemens, Germany). The 25(OH)D was detected by investigators who were blinded to phenotypic and genotypic data, and measured with chemiluminescenceassay (SiemensADVIA Centaur XP, Germany). The detection range of the assay for 25(OH)D is 10.5-375 nmol/L [19].

### MS definition

MS was determined based on the 2005 revision of the criteria from the National Cholesterol Education Program Adult Treatment Panel III [20]. A person with MS must have: abdominal obesity (waist circumference: male ≥90 cm, female ≥80 cm, or BMI is ≥30 kg/m^2^) plus at least two of the following criteria: (i) Raised TG > 1.7 mmol/L, or treatment for this dyslipidemia; (ii) Reduced HDL-C < 1.03 mmol/L in men or < 1.29 mmol/L in women, or treatment for this dyslipidemia; (iii) Raised BP: systolic BP ≥130 or diastolic BP ≥85 mmHg, or treatment for hypertension; (iv) Raised FPG ≥5.6 mmol/L or a history of type 2 diabetes.

### Genotyping, genetic loci selection and genetic risk score construction

DNA was extracted from peripheral white blood cells using a blood genomic DNA extraction kit (DP603, TIANGEN BIOTECH CO, LTD, Beijing, China) on an automated nucleic acid extraction instrument (YOSE-S32, TIANGEN BIOTECH CO, LTD, Beijing, China). Specific assays were designed using Geneious Pro (v4.8.3) (https://www.geneious.com/). Mass determination was performed using the JUNO and data acquisition was completed with Fluidigm SNP Genotyping Analysis v4.1.3 software (Fluidigm Corporation, South San Francisco, California, USA). Genotyping was performed blinded to 25(OH)D and cardiometabolic profile. Call rates for all SNPs were greater than 98%.

The four vitamin D-related SNPs [*DHCR7* (related to vitamin D synthesis) rs12785878, *CYP2R1* (hepatic 25-hydroxylation) rs10741657, *GC* (transport) rs2282679, and *CYP24A1* (catabolism) rs6013897] were chosen on the basis of a recent MR study containing Asian participants [21]. These SNPs were also used in previous mendelian analyses in Chinese [22, 23]. They all achieved a genome-wide significance level in genome-wide association studies (*P* < 5 × 10^− 8^) and showed no sign of linkage disequilibrium (r^2^ = 0). The full list of each SNP was displayed in Additional file 1:Table S1.

### Statistical analysis

All statistical analyses were undertaken using IBM SPSS Statistics, Version 22 (IBM Corporation, Armonk, NY, USA). All analyses were two-sided. A *P* value < 0.05 indicated significance. Continuous and categorical variables were expressed as the mean ± standard deviation (SD) and as percentages (%), respectively. Additive models with MS and related metabolic traits as outcomes were adjusted for age, sex, urban/rural residence, economic status, current smoking, waist circumference, diabetes, hypertension, HDL-cholesterol and ln (triglycerides); models with 25(OH)D concentration as the outcome were additionally adjusted for season of sampling.

The additive genetic model for each SNP was used to construct GRS. Each SNP was coded 0–2 based on the number of effect alleles and then multiplied by the β value from the previous study [21], followed by summing the four values. In one SNP, those missing ones were assigned the median score (0, 1 or 2). We not only calculated GRS_combined_ containing all four SNPs. Additionally, we did separate mendelian randomization analyses using GRSs for SNPs for 25(OH)D synthesis (*DHCR7* and *CYP2R1*) and metabolism (*GC* and *CYP24A1*).

We first assessed the association of each SNP with 25(OH)D concentration using linear regression. Then, we examined associations of each SNP with metabolic syndrome and its components. Serum TG was logarithmically transformed prior to analysis.

For the main mendelian randomization analysis, linear regression analyses were used to determine the association estimates (**β**_**ZX**_) of GRS_combined_, GRS_synthesis_ and GRS_metabolism_ with 25(OH)D. We also assessed the strength of associations using *F*-statistics [*F* = (R^2^*(n-2))/(1-R^2^)] [24]. *F* values greater than 10 were regarded as strong enough for MR analysis [25]. Then, we examined the association (**β**_**ZY**_) of GRS_combined_, GRS_synthesis_ and GRS_metabolism_ with the phenotypes (MS and its components) using linear regression for continuous variables and logistic regression for binary variables.

To explore the observational association (**β**_**XY**_) of 25(OH)D on phenotypes (MS and its components), We generated linear regression models for continuous outcomes (e.g., waist circumference) and logistic regression models for binary outcomes (e.g., MS). Effect estimates were presented per 10 nmol/L increase in 25(OH)D.

We used the GRSs as the IVs to estimate the causal effect of 25(OH)D on the same outcome diseases and measures. We calculated IV estimates of genetically determined β coefficients or odds ratios (OR) with the Wald-type estimator [25]. For continuous outcomes (waist circumference, FPG, lnTG, HDL and blood pressure), the computational formula was β_IV(VD-outcome)_ = β_GRS-outcome_ / β_GRS-VD_. For dichotomous outcomes (central obesity, raised fasting plasma glucose, triglyceride and blood pressure, reduced HDL and MS), the computational formula was OR_IV(VD-outcome)_ = exp. (ln (OR_GRS-outcome_) / β_GRS-VD_).

In the sensitivity analyses, we used another IV calculating method, a two-stage regression estimator to calculate causal β coefficients or ORs per 10 nmol/L increase in 25(OH)D [26]. In the first stage, a linear regression of 25(OH)D on GRSs was used to generate 25(OH)D fitted values. In the second stage, The predicted 25(OH)D values from the first stage were used for linear and logistic regression analyses with the metabolic traits and MS as the dependent variable.

## Results

The study comprised 10,655 participants from general population who had detailed metabolic profiles measured and information on gene score for predisposition to decreased 25(OH)D. In them, 60.0% were women, and all were of Asian descent. The mean age was 54.9 (SD 12.9) y, and mean waist circumference and 25(OH)D was 81 (SD 10) cm and 40.6 (SD 12.8) nmol/L at the time of clinical assessment. The prevalence of MS was 27.8%.

### Association of SNPs with 25(OH)D and MS

We tested the association of four vitamin D-related SNPs with 25(OH)D, MS and related metabolic traits (Additional file 1: Table S2). In the four vitamin D-related SNPs, all four effect alleles showed an inverse linear relationship with 25(OH)D level and two had significant associations (*GC* and *DHCR*) after adjusting for age, sex, urban/rural residence, economic status and current smoking. None of the 25(OH)D related SNPs were significantly associated with MS or its metabolic traits including waist circumference, FPG, lnTG, HDL, blood pressure.

### Study characteristics according to GRS_combined_

**Fig. 2:**
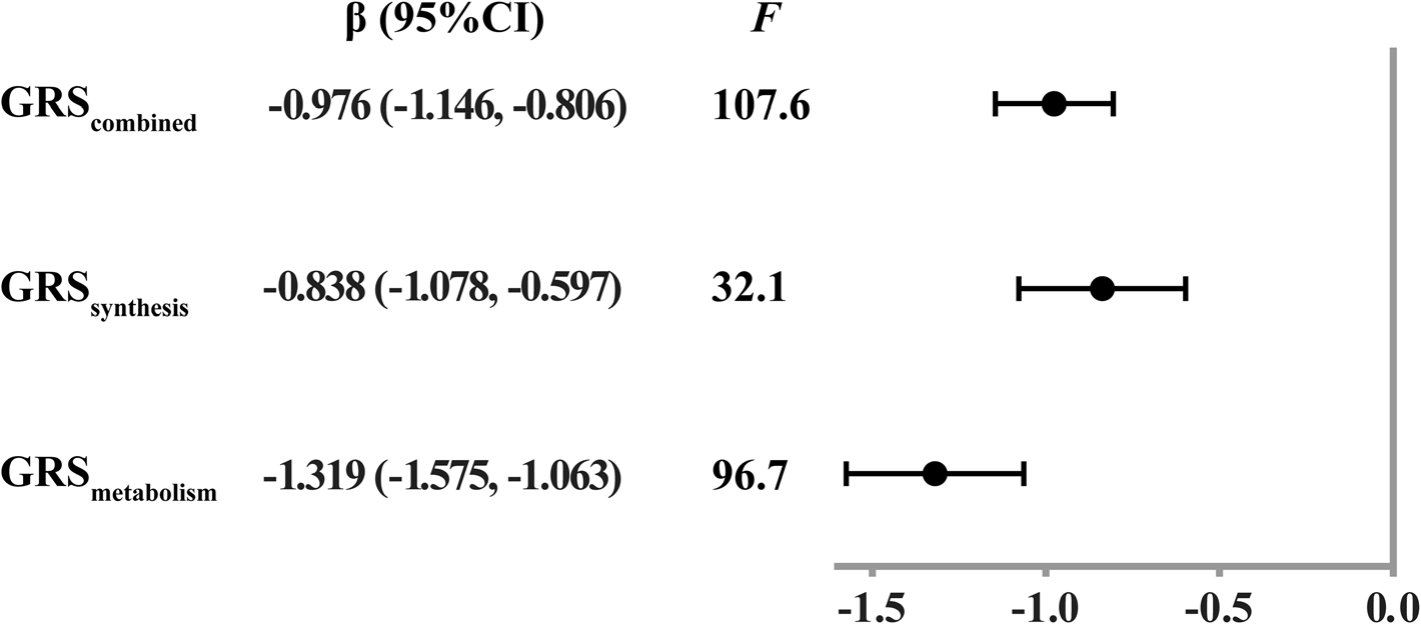
Association between VD_GRS and 25-hydroxyvitamin D

As expected, with increasing quartiles of VD_GRS, 25(OH)D concentrations significantly decreased in both men and women (Table 1). Univariable linear regression demonstrated a strong relationship between GRSs and 25(OH)D level (β_ZX_). Each 1 unit increment of GRS_combined_, GRS_synthesis_ and GRS_metabolism_ was associated with a decrease of 0.976 (95% CI 0.806, 1.146), 0.838 (95% CI 0.597, 1.078) and 1.319 (95%CI 1.063, 1.575) nmol/L in 25(OH)D levels, respectively (Fig. 2). The *F*-statistic of GRS_combined_, GRS_synthesis_ and GRS_metabolism_ was 107.6, 32.1 and 96.7, respectively, indicating strong IVs for MR.

**Table 1:** Characteristics of study participants according to the weighted vitamin D genetic risk score (GRS_combined_) stratified by gender (*n* = 10655)

Characteristic	GRS_combined_				*P* for trend
	Quartile 1	Quartile 2	Quartile 3	Quartile 4	
Men					
GRS_combined_	≤2.480	2.481–3.520	3.521–4.640	≥4.641	
N	1072	1066	995	952	
Age, yr	55.5 (13.2)	55.5 (13.2)	55.7 (13.1)	55.8 (13.0)	0.547
25(OH)D, mmol/L	45.3 (14.1)	44.0 (13.3)	42.7 (13.6)	41.5 (12.7)	< 0.001
Fasting plasma glucose, mmol/L	5.67 (1.43)	5.72 (1.54)	5.76 (1.63)	5.87 (1.81)	0.005
Body mass index, kg/m^2^	25.0 (3.4)	24.9 (3.4)	25.0 (3.6)	25.0 (3.5)	0.719
Waist circumference, cm	85.1 (9.9)	85.2 (9.2)	85.1 (9.5)	85.3 (9.5)	0.714
Triglycerides, mmol/L	1.85 (1.93)	1.85 (1.95)	1.91 (1.93)	1.91 (1.60)	0.345
High-density lipoprotein, mmol/L	1.32 (0.32)	1.33 (0.32)	1.33 (0.33)	1.31 (0.31)	0.566
Systolic blood pressure, mmHg	135 (21)	134 (20)	135 (21)	134 (21)	0.731
Diastolic blood pressure, mmHg	82 (13)	82 (13)	82 (13)	82 (13)	0.224
Current smoker, %	44.1	44.7	45.2	46.9	0.712
Central obesity, %	31.8	32.6	32.1	33.2	0.579
Raised fasting plasma glucose, %	36.6	38.1	39.8	42.0	0.006
Raised triglycerides, %	42.1	44.4	43.9	42.9	0.796
Reduced high-density lipoprotein, %	25.3	26.0	26.3	28.4	0.105
Raised blood pressure, %	67.8	68.9	68.6	65.7	0.286
Metabolic syndrome, %	21.9	25.0	24.4	23.7	0.416
Rural/urban residence, %	49.3/50.7	49.2/50.8	49.7/50.3	48.9/51.1	0.936
Low/high econmic status, %	33.9/66.1	34.2/65.8	35.7/64.3	36.7/63.3	0.129
Women					
GRS_combined_	≤2.440	2.441–3.480	3.481–4.640	≥4.641	
N	1601	1189	1493	1418	
Age, yr	54.0 (12.8)	54.4 (12.9)	54.5 (12.8)	54.7 (12.3)	0.120
Premenopausal women, %	47.7	47.4	46.1	45.3	0.161
25(OH)D, mmol/L	40.3 (12.5)	38.5 (11.9)	38.7 (12.1)	37.4 (11.2)	< 0.001
Fasting plasma glucose, mmol/L	5.58 (1.43)	5.62 (1.43)	5.61 (1.46)	5.57 (1.32)	0.868
Body mass index, kg/m^2^	24.3 (3.6)	24.5 (3.5)	24.4 (3.7)	24.4 (3.7)	0.668
Waist circumference, cm	78.3 (9.8)	78.9 (9.8)	78.6 (10.1)	78.5 (9.6)	0.592
Triglycerides, mmol/L	1.52 (1.05)	1.59 (1.18)	1.48 (1.06)	1.55 (1.07)	0.985
High-density lipoprotein, mmol/L	1.44 (0.31)	1.45 (0.31)	1.47 (0.32)	1.45 (0.32)	0.238
Systolic blood pressure, mmHg	131 (22)	132 (21)	131 (22)	132 (22)	0.258
Diastolic blood pressure, mmHg	77 (13)	78 (13)	78 (13)	78 (13)	0.424
Current smoker, %	1.9	2.0	2.5	2.0	0.487
Central obesity, %	43.8	46.5	45.2	47.0	0.150
Raised fasting plasma glucose, %	33.4	35.4	33.8	34.1	0.892
Raised triglycerides, %	36.1	36.3	31.3	35.7	0.244
Reduced high-density lipoprotein, %	39.8	38.0	34.6	38.1	0.110
Raised blood pressure, %	56.6	58.9	56.0	57.7	0.905
Metabolic syndrome, %	30.0	32.4	29.8	31.2	0.809
Rural/urban residence, %	44.9/55.1	45.8/54.2	47.2/52.8	46.9/53.1	0.193
Low/high econmic status, %	32.5/67.5	31.7/68.3	32.4/67.6	32.5/67.5	0.910

The data are summarized as the mean (SD) for continuous variables or as a numerical proportion for categorical variables. *P* for trend was calculated by ANOVA and chi-square tests. 25(OH)D, 25-hydroxyvitamin D; GRS, genetic risk score

The calculated risk score GRS_combined_ was neither consistently associated with any of the investigated MS components such as blood lipids, BP, or waist circumference, nor with other risk factors such as age, sex, smoking, and economic status, except for an association with FPG in men. There was also no association with the prevalence of MS.

### Associations of 25(OH)D and GRSs with MS

Regarding the cross-sectional associations of 25(OH)D with metabolic syndrome traits (continuous variables) (β_XY_), lnTG was negatively associated with 25(OH)D (0.059 decrease per 10 nmol/L increase in 25(OH)D), but both systolic and diastolic blood pressure showed positive association with 25(OH)D. Waist circumference, FPG and HDL showed no significant association (Table 2). The cross-sectional associations with metabolic syndrome components (dichotomous variables) (OR_XY_) were coherent with metabolic syndrome traits (Table 3). Metabolic syndrome was negatively associated with 25(OH)D (OR 0.921, 95% CI 0.888, 0.954).

**Table 2:** Causal coefficients from MR analysis for the associations of 25(OH)D with metabolic syndrome components (continuous variables)

	β_XY_ [per 10 nmol/L increase in 25(OH)D]	GRS_combined_		GRS_synthesis_		GRS_metabolism_	
		β_ZY_ [per 1 unit increase in GRS_combined_]	β_IV_ [per 10 nmol/L increase in 25(OH)D]	β_ZY_ [per 1 unit increase in GRS_synthesis_]	β_IV_ [per 10 nmol/L increase in 25(OH)D]	β_ZY_ [per 1 unit increase in GRS_metabolism_]	β_IV_ [per 10 nmol/L increase in 25(OH)D]
Waist circumference	0.128 (−0.005, 0.262)	−0.038 (−0.155, 0.080)	0.388 (− 0.814,1.590)	− 0.061 (− 0.227, 0.104)	0.726 (−1.245, 2.697)	− 0.004 (− 0.180, 0.172)	0.030 (−1.306, 1.366)
Fasting plasma glucose	− 0.012 (− 0.034, 0.011)	0.017 (− 0.002, 0.037)	−0.174 (− 0.376, 0.029)	0.027 (− 0.001, 0.055)	−0.321 (− 0.660, 0.018)	0.004 (− 0.026, 0.034)	−0.030 (− 0.253, 0.193)
ln(triglycerides)	**− 0.059 (− 0.067, − 0.051)**	0.002 (− 0.004, 0.009)	−0.020 (− 0.080, 0.040)	0.002 (− 0.007, 0.012)	−0.024 (− 0.141, 0.093)	0.003 (− 0.007, 0.013)	−0.023 (− 0.097, 0.051)
High-density lipoprotein	− 0.003 (− 0.008, 0.001)	0.001 (− 0.003, 0.005)	−0.010 (− 0.050, 0.030)	0.001 (− 0.005, 0.007)	−0.012 (− 0.082, 0.058)	0.001 (− 0.005, 0.007)	−0.008 (− 0.053, 0.037)
Systolic blood pressure	**0.468 (0.168, 0.768)**	0.018 (−0.246, 0.283)	− 0.184 (−2.884, 2.516)	0.064 (− 0.309, 0.437)	−0.762 (−5.200, 3.676)	−0.054 (− 0.451, 0.344)	0.409 (− 2.606, 3.424)
Diastolic blood pressure	**0.287 (0.092, 0.481)**	− 0.006 (− 0.177, 0.166)	0.061 (− 1.679, 1.801)	−0.014 (− 0.256, 0.228)	0.167 (− 2.703, 3.037)	0.007 (− 0.251, 0.264)	−0.053 (− 1.998, 1.892)

Data are presented as regression coefficients (95% confidence interval). *25(OH)D* 25-hydroxyvitamin D, *GRS* genetic risk score, *MR* mendelian randomization

The bold number represents significance (*P* < 0.05)

The model was adjusted for age, sex, urban/rural residence, economic status, current smoking, waist circumference, diabetes, hypertension, HDL-cholesterol, ln(triglycerides)

**Table 3:** Causal odds ratios from MR analysis for the associations of 25(OH)D with metabolic syndrome components (dichotomous variables) and metabolic syndrome

	OR_XY_ [per 10 nmol/L increase in 25(OH)D]	GRS_combined_		GRS_synthesis_		GRS_metabolism_	
		OR_ZY_ [per 1 unit increase in GRS_combined_]	OR_IV_ [per 10 nmol/L increase in 25(OH)D]	OR_ZY_ [per 1 unit increase in GRS_synthesis_]	OR_IV_ [per 10 nmol/L increase in 25(OH)D]	OR_ZY_ [per 1 unit increase in GRS_metabolism_]	OR_IV_ [per 10 nmol/L increase in 25(OH)D]
Central obesity	1.023 (0.987, 1.061)	1.014 (0.982, 1.047)	0.867 (0.629, 1.195)	1.008 (0.964, 1.055)	0.909 (0.532, 1.556)	1.024 (0.975, 1.075)	0.834 (0.574, 1.210)
Raised fasting plasma glucose	1.016 (0.981, 1.052)	1.027 (0.994, 1.060)	0.767 (0.555, 1.061)	**1.047 (1.001, 1.095)**	**0.578 (0.321, 0.980)**	0.997 (0.951, 1.046)	1.034 (0.717, 1.461)
Raised triglyceride	**0.833 (0.803, 0.864)**	0.966 (0.965, 1.027)	1.042 (0.757, 1.435)	0.988 (0.945, 1.033)	1.154 (0.674, 1.976)	1.008 (0.961, 1.057)	0.941 (0.658, 1.344)
Reduced high-density lipoprotein	1.006 (0.969, 1.044)	0.991 (0.959, 1.024)	1.096 (0.780, 1.540)	0.983 (0.939, 1.030)	1.224 (0.697, 2.149)	1.003 (0.955, 1.053)	0.978 (0.674, 1.416)
Raised blood pressure	**1.043 (1.004, 1.084)**	0.978 (0.946, 1.011)	1.251 (0.889, 1.761)	0.978 (0.933, 1.025)	1.300 (0.739, 2.286)	0.975 (0.928, 1.025)	1.209 (0.832, 1.754)
Metabolic syndrome	**0.921 (0.888, 0.954)**	0.977 (0.966, 1.030)	1.031 (0.749, 1.420)	0.994 (0.950, 1.041)	1.074 (0.628, 1.837)	1.002 (0.955, 1.052)	0.985 (0.680, 1.428)

Data are presented as odds ratios (95% confidence interval). *25(OH)D* 25-hydroxyvitamin D, *GRS* genetic risk score, *MR* mendelian randomization

The bold number represents significance (*P* < 0.05)

The model was adjusted for age, sex, urban/rural residence, economic status, current smoking, waist circumference, diabetes, hypertension, HDL-cholesterol, ln(triglycerides). For metabolic syndrome, the model was adjusted for age, sex, urban/rural residence, economic status and current smoking

Regarding β_ZY_, one unit increase in VD_GRS, VD_GRS_metabolism_, and VD_GRS_synthesis_ was not significantly associated with metabolic syndrome traits (continuous variables), except that GRS_combined_ and GRS_synthesis_ showed marginally significant association with FPG (*P* = 0.086 and 0.061, respectively) (Table 2). For dichotomous variables, GRS_synthesis_ showed significant association with raised FPG (OR 1.047, 95% CI 1.001, 1.095) and other associations were in accordance with those with continuous variables (Table 3).

### 25(OH)D and MS: the MR analysis

The causal effects of 25(OH)D on the MS and its traits were analyzed using MR (Tables 2 and 3). Although association was found between 25(OH)D concentration and lnTG as well as systolic and diastolic blood pressure, the 95% confidence intervals (95% CIs) of all calculated β_IV_s gave no evidence for rejecting the null hypothesis of no association, except that 25(OH)D genetically determined by GRS_combined_ and GRS_synthesis_ showed marginally significant association with FPG (β_IV_ -0.174, 95% CI -0.376, 0.029, *P* = 0.086 and β_IV_ -0.321 95% CI -0.660, 0.018, *P* = 0.061, respectively). Furthermore, MR analysis also found the association for raised FPG with OR_IV_ of 0.578 (95% CI 0.321, 0.980) per 10 nmol/L GRS_synthesis_ determined increase of 25(OH)D levels, but GRSs were not causally associated with the central obesity, raised TG, reduced HDL and raised blood pressure. Finally, the null association persisted in 25(OH)D and MS.

### Sensitivity analysis

We also used a two-stage regression estimator to calculate the β_IV_s and OR_IV_s per 10 nmol/L increase in 25(OH)D (Additional file 1: Tables S3 and S4). The results were rather similar to those using Wald-type estimator. The only significant association was also observed between 25(OH)D genetically determined by GRS_synthesis_ and raised FPG (OR 0.546, 95% CI 0.302, 0.984 per 10 nmol 25(OH)D increase).

## Discussion

In this community-dwelling sample of Chinese adults, inverse cross-sectional associations between 25(OH)D and MS were observed after multivariate adjustment. However, the results of MR analysis provide no evidence that genetically lowered 25(OH)D is associated with increased risk of MS and its metabolic traits. The current study is, to our knowledge, the first MR study to investigate the effect of vitamin D on MS.

Most studies to date have reported cross-sectional associations of low vitamin D status with MS and its metabolic traits [4–6]. Although some studies indicated that optimization of vitamin D status could reduce some proatherogenic inflammatory markers and improve cardiometabolic profile [9, 27], meta-analyses of RCTs have consistently demonstrated no beneficial effects of vitamin D supplementation on cardiometabolic risk factors such as blood pressure [28], lipid profile [29] and cardiovascular diseases [30]. In line with our findings, two recent MR studies found that genetically reduced 25(OH)D is not associated with ischemic heart disease [31], coronary artery disease [32] and cardiovascular mortality [33].

Several possible reasons may explain the paradox of beneficial findings of observational studies versus disappointing results of RCTs and MR studies. First, residual confounding may account for a large part of the discrepancy. Though the association was significant in the cross-sectional setting, it may often still be a biased estimate, due to an inadequate control of potential confounders. Take outdoor physical activity for example: it is associated with cardiometabolic health [34] and solar exposure, which in turn influences vitamin D status. Second, low vitamin D status is an outcome, rather than a cause, of poor health (ie, reverse causation) [35]. Bi-directional MR studies suggest that the cardiometabolic risk factors, such as low density lipoprotein cholesterol, high remnant cholesterol and obesity may causally induce lower vitamin D status, whereas a lower 25(OH)D is unlikely to increase blood cholesterol and BMI [24, 36]. That is, low serum 25(OH)D might be a consequence of a predisposition to cardiometabolic disorders rather than a cause of cardiometabolic diseases.

Interestingly, we observed that the synthesis allele score was significantly associated with increased risk of raised FPG, whereas the allele score for 25(OH)D metabolism was not. As we know, the DHCR7 gene converts 7-dehydrocholesterol to cholesterol, providing a substrate for vitamin D production. CYP2R1 plays a key role in 25-hydroxylation of vitamin D in the liver [37]. Thus, we may speculate that low vitamin D status via liver conversion from all sources (sunlight exposure, diet, and supplementation) is associated with raised FPG. Of note, genetically variants in DHCR7 and CYP2R1 have previously been demonstrated to be associated with increased risk of type 1 diabetes [38]. Specifically, GC encodes the vitamin D binding protein, which binds and transports 80–90% of 25(OH)D to target organs. CYP24A1 is responsible for degradation of 25(OH)D [39]. Consequently, components included in the metabolism allele score are of importance for the transfer and clearance of 25(OH)D and could provide insights into the influence of vitamin D metabolism on FPG. Nevertheless, the use of metabolism allele score as a formal instrument in MR analyses is susceptible to problems with regard to quantification of expected associations, pleiotropic effects, and metabolic feedback loops associated with the clearance of vitamin D-related metabolites by CYP24A1 [40]. Investigations with the vitamin D metabolism allele score were accordingly exploratory only. Last, we cannot rule out the possibility that the findings could have arisen by chance and should be replicated in further MR studies.

Considering cardiometabolic disease is the leading cause of death globally [41] and the sale of vitamin D supplements is increasing annually in older populations worldwide [42], our MR findings are relevant to clinical care. MR analyses using DHCR7, GC, CYP24A1, and CYP2R1 as IVs have been implemented in the past 5 years [23, 31, 40, 43]. We and others recently provided evidence from MR that genetically reduced vitamin D levels do not increase the risk of non-alcoholic fatty liver disease [23], type 2 diabetes mellitus [43] or ischemic heart disease [31], but might increase the risk of high blood pressure [40]. The present study also showed no evidence of a causal association between vitamin D and MS. In this regard, most association between vitamin D and cardiometabolic profile that seemed promising in observational studies did not withstand rigorous testing for causation in MR studies. Hence, vitamin D supplementation may not be a clinical priority in cardiometabolic disorders prevention.

The strength of our study was the relatively large sample of community-dwelling population, the MR study design, the detailed information on covariates and a created VD_GRS representing the combined effect of the established common genetic variations of 25(OH)D as the IV. In addition, the SNPs we used are a robust proxy for circulating levels of 25(OH)D and have well understood roles in vitamin D synthesis and metabolic pathway. Lastly, we did not detect any violations of the assumptions underlying MR as far as they could be tested.

However, there are several limitations we should acknowledge. Firstly, all participants were of Asian descent, and thus, our findings may not be necessarily generalizable to other ethnicities. Secondly, we created our VD_GRS only on the basis of common variants, which was considered to represent limited amount of variance in 25(OH)D. We were unable to assess the potential contribution of rare variants. Lastly, we have no data of 1,25-dihydroxyvitamin D, the active form of vitamin D metabolite leading to biological effects.

## Conclusion

We found no evidence that genetically reduced 25(OH)D is associated with increased risk of MS and its metabolic traits. This suggests that the observed association of increased 25(OH)D with favorable metabolic profile may not represent a causal relationship, but more likely is owing to uncontrolled confounding or owing to reverse causation.

## Supplementary information

**Additional file 1: Table S1.** Information of each SNP in GRS. **Table S2.** The association of each individual SNP with vitamin D, metabolic syndrome and its component. **Table S3.** Causal coefficients from MR analysis for the associations of 25(OH)D with metabolic syndrome components, using two-stage regression estimator. **Table S4.** causal odds ratios from MR analysis for the associations of 25(OH)D with metabolic syndrome components, using two-stage regression estimator.

## Data Availability

The datasets during and/or analyzed during the current study are available from the corresponding author on reasonable request.

## Abbreviations

25(OH)D: 25-hydroxyvitamin D
BMI: Body mass index
FPG: Fasting plasma glucose
GRS: Genetic risk score
HDL: High-density lipoprotein
IV: Instrumental variable
MR: Mendelian randomization
MS: Metabolic syndrome
OR: Odds ratio
RCT: Randomized controlled trial
SNP: Single nucleotide polymorphism
TG: Triglycerides
