# Supplementary material for: Association of 25-hydroxyvitamin D with cardiometabolic risk factors and metabolic syndrome: a mendelian randomization study
Source: Nutr J. 2019 Oct 28;18:61. doi: 10.1186/s12937-019-0494-7 (PMC6819483; doi:10.1186/s12937-019-0494-7)
Supplement: Supplementary file 1 — Additional file 1: Table S1. Information of each SNP in GRS. Table S2. The association of each individual SNP with vitamin D, metabolic syndrome and its component. Table S3. Causal coefficients from MR analysis for the associations of 25(OH)D with metabolic syndrome components, using two-stage regression estimator. Table S4. causal odds ratios from MR analysis for the associations of 25(OH)D with metabolic syndrome components, using two-stage regression estimator. [file 12937_2019_494_MOESM1_ESM.docx]

Table S1 Information of each SNP in GRS

| Gene | Chromosome | Position | SNP | Effect allele | EAF | *X^2^* | *P* for HWE |
| --- | --- | --- | --- | --- | --- | --- | --- |
| GC | 4 | 71742666 | rs2282679 | G | 0.32 | 0.32 | >0.05 |
| CYP2R1 | 11 | 14893332 | rs10741657 | G | 0.65 | 3.06 | >0.05 |
| DHCR7 | 11 | 71456403 | rs12785878 | G | 0.55 | 3.56 | >0.05 |
| CYP24A1 | 20 | 54125940 | rs6013897 | A | 0.16 | 9.57 | 0.001<*P*<0.005 |

EAF, effect allele frequency; GRS, genetic risk score; HWE, Hardy-Weinberg equilibrium; SNP, single nucleotide polymorphism.

Table S2 The association of each individual SNP with vitamin D, metabolic syndrome and its components

|  | rs2282679 | | rs10741657 | | rs12785878 | | rs6013897 | |
| --- | --- | --- | --- | --- | --- | --- | --- | --- |
|  | B(SE) | *P* | B(SE) | *P* | B(SE) | *P* | B(SE) | *P* |
| 25(OH)D | -1.917(0.179) | <0.001 | -0.315(0.176) | 0.07 | -1.284(0.170) | <0.001 | -0.281(0.223) | 0.21 |
| Waist circumference | 0.095(0.138) | 0.49 | -0.107(0.136) | 0.43 | -0.047(0.131) | 0.72 | -0.309(0.172) | 0.07 |
| Fasting plasma glucose | 0.016(0.021) | 0.47 | 0.028(0.021) | 0.18 | 0.012(0.020) | 0.55 | -0.015(0.027) | 0.56 |
| ln(triglycerides) | -0.002(0.008) | 0.83 | 0.000(0.008) | 0.99 | -0.003(0.007) | 0.72 | 0.009(0.010) | 0.34 |
| High-density lipoprotein | -0.003(0.005) | 0.49 | 0.000(0.005) | 0.91 | 0.002(0.004) | 0.72 | 0.009(0.006) | 0.13 |
| Systolic blood pressure | 0.045(0.289) | 0.88 | 0.118(0.283) | 0.68 | -0.040(0.273) | 0.88 | -0.211(0.360) | 0.56 |
| Diastolic blood pressure | 0.014(0.188) | 0.94 | -0.016(0.185) | 0.93 | -0.072(0.178) | 0.69 | 0.038(0.234) | 0.87 |
|  |  |  |  |  |  |  |  |  |
|  | OR (95%CI) | *P* | OR (95%CI) | *P* | OR (95%CI) | *P* | OR (95%CI) | *P* |
| Central obesity | 1.046(0.982, 1.113) | 0.16 | 0.995(0.936, 1.058) | 0.87 | 1.011(0.952, 1.072) | 0.73 | 0.950(0.878, 1.027) | 0.20 |
| Raised fasting plasma glucose | 1.019(0.956, 1.085) | 0.56 | 1.044(0.981, 1.111) | 0.17 | 1.031(0.971, 1.094) | 0.31 | 0.946(0.874, 1.023) | 0.16 |
| Raised triglyceride | 0.996(0.937, 1.058) | 0.89 | 1.006(0.948, 1.067) | 0.85 | 0.958(0.905, 1.014) | 0.14 | 1.010(0.937, 1.089) | 0.79 |
| Reduced high-density lipoprotein | 1.022(0.960, 1.088) | 0.49 | 1.012(0.951, 1.076) | 0.71 | 0.966(0.910, 1.024) | 0.25 | 0.979(0.905, 1.024) | 0.60 |
| Raised blood pressure | 0.973(0.911, 1.040) | 0.42 | 0.962(0.901, 1.026) | 0.24 | 1.000(0.939, 1.064) | 0.99 | 0.992(0.913, 1.077) | 0.84 |
| Metabolic syndrome | 1.010(0.944, 1.080) | 0.78 | 1.025(0.960, 1.096) | 0.46 | 0.969(0.909, 1.033) | 0.33 | 0.983(0.903, 1.069) | 0.68 |

Data are expressed as unstandardized coefficients (standard error) or odds ratios (95% confidence interval). Multiple linear regression was performed. The model was adjusted for age, sex, urban/rural residence, economic status and current smoking.

Table S3 causal coefficients from MR analysis for the associations of 25(OH)D with metabolic syndrome components, using two-stage regression estimator

|  | GRS_combined_ |  | GRS_synthesis_ |  | GRS_metabolism_ |
| --- | --- | --- | --- | --- | --- |
|  | β_IV_ per 10nmol/L increase in 25(OH)D |  | β_IV_ per 10nmol/L increase in 25(OH)D |  | β_IV_ per 10nmol/L increase in 25(OH)D |
| Waist circumference | 0.403(-0.854, 1.659) |  | 0.806(-1.374, 2.986) |  | 0.027(-1.295, 1.350) |
| Fasting plasma glucose | -0.186(-0.399, 0.026) |  | -0.353(-0.722, 0.016) |  | -0.031(-0.255, 0.193) |
| ln(triglycerides) | -0.026(-0.099, 0.047) |  | -0.030(-0.156, 0.097) |  | -0.022(-0.099, 0.055) |
| High-density lipoprotein | -0.010(-0.053, 0.033) |  | -0.012(-0.087, 0.063) |  | -0.008(-0.054, 0.037) |
| Systolic blood pressure | -0.198(-3.032, 2.637) |  | -0.845(-5.763, 4.074) |  | 0.404(-2.580, 3.387) |
| Diastolic blood pressure | 0.061(-1.776, 1.899) |  | 0.182(-3.006, 3.370) |  | -0.051(-1.985, 1.883) |

Data are presented as regression coefficient (95% confidence interval). 25(OH)D, 25-hydroxyvitamin D; GRS, genetic risk score.

The model was adjusted for age, sex, urban/rural residence, economic status, current smoking, waist circumference, diabetes, hypertension, HDL-cholesterol, ln(triglycerides).

Table S4 causal odds ratios from MR analysis for the associations of 25(OH)D with metabolic syndrome components, using two-stage regression estimator

|  | GRS_combined_ |  | GRS_synthesis_ |  | GRS_metabolism_ |
| --- | --- | --- | --- | --- | --- |
|  | OR_IV_ per 10nmol/L increase in 25(OH)D |  | OR_IV_ per 10nmol/L increase in 25(OH)D |  | OR_IV_ per 10nmol/L increase in 25(OH)D |
| Central obesity | 0.865(0.613, 1.221) |  | 0.895(0.492, 1.626) |  | 0.838(0.583, 1.205) |
| Raised fasting plasma glucose | 0.755(0.538, 1.061) |  | **0.546(0.302, 0.984)** |  | 1.022(0.714, 1.462) |
| Raised triglyceride | 1.049(0.748, 1.471) |  | 1.178(0.656, 2.118) |  | 0.940(0.658, 1.344) |
| Reduced high-density lipoprotein | 1.100(0.775, 1.563) |  | 1.246(0.678, 2.288) |  | 0.980(0.678, 1.418) |
| Raised blood pressure | 1.265(0.886, 1.807) |  | 1.335(0.718, 2.483) |  | 1.205(0.828, 1.753) |
| Metabolic syndrome | 1.027(0.727, 1.452) |  | 1.077(0.590, 1.965) |  | 0.984(0.683, 1.417) |

Data are presented as regression coefficient (95% confidence interval). 25(OH)D, 25-hydroxyvitamin D; GRS, genetic risk score.

The model was adjusted for age, sex, urban/rural residence, economic status, current smoking, waist circumference, diabetes, hypertension, HDL-cholesterol and ln(triglycerides).
